# Supplementary material for: Displacement efficiency of alternative energy and trans-provincial imported electricity in China
Source: Nat Commun. 2017 Feb 17;8:14590. doi: 10.1038/ncomms14590 (PMC5321750; doi:10.1038/ncomms14590)
Supplement: Supplementary Information — Supplementary Figures, Supplementary Tables and Supplementary References [file ncomms14590-s1.pdf]

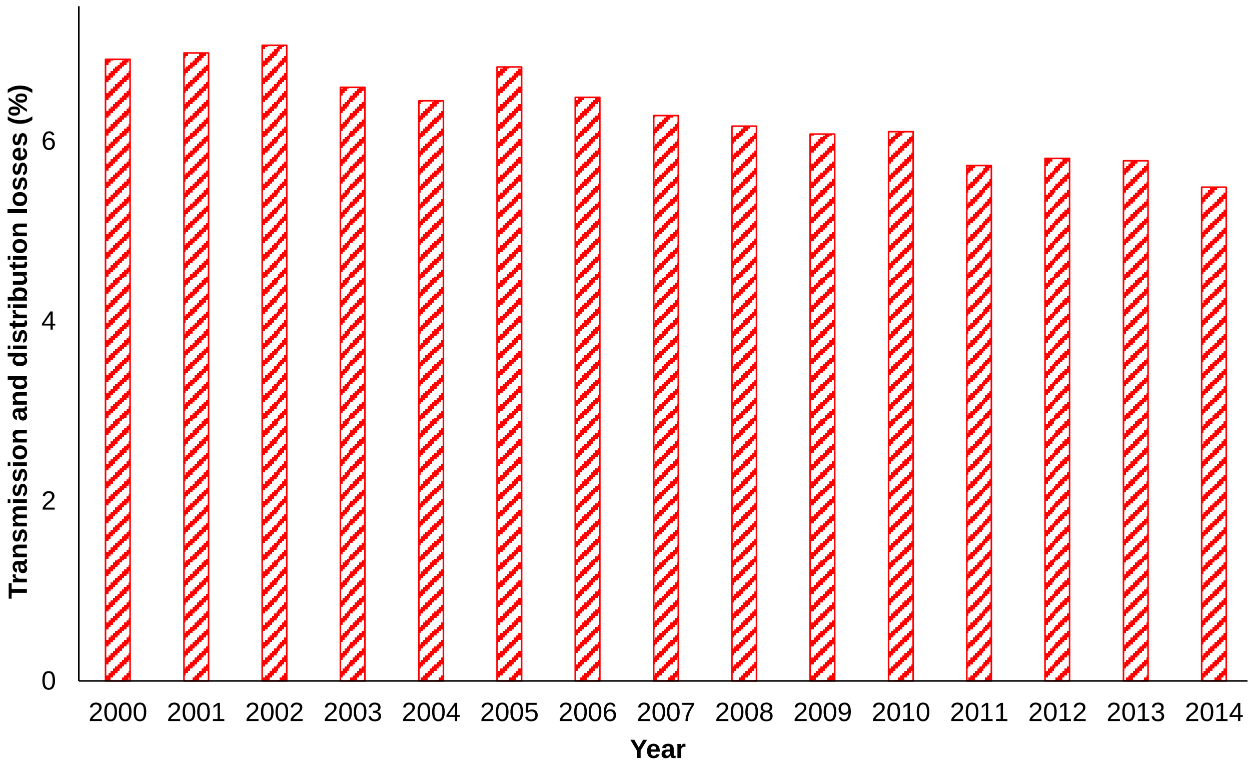

Supplementary Figure 1 | Average electricity transmission and distribution losses in China between 2000 and 2014. China's average electricity transmission and distribution losses in recent years were close to those of the United States (about 6%). Data from the electricity balance table of the National Bureau of Statistics (http://data.stats.gov.cn/easyquery.htm?cn=C01).

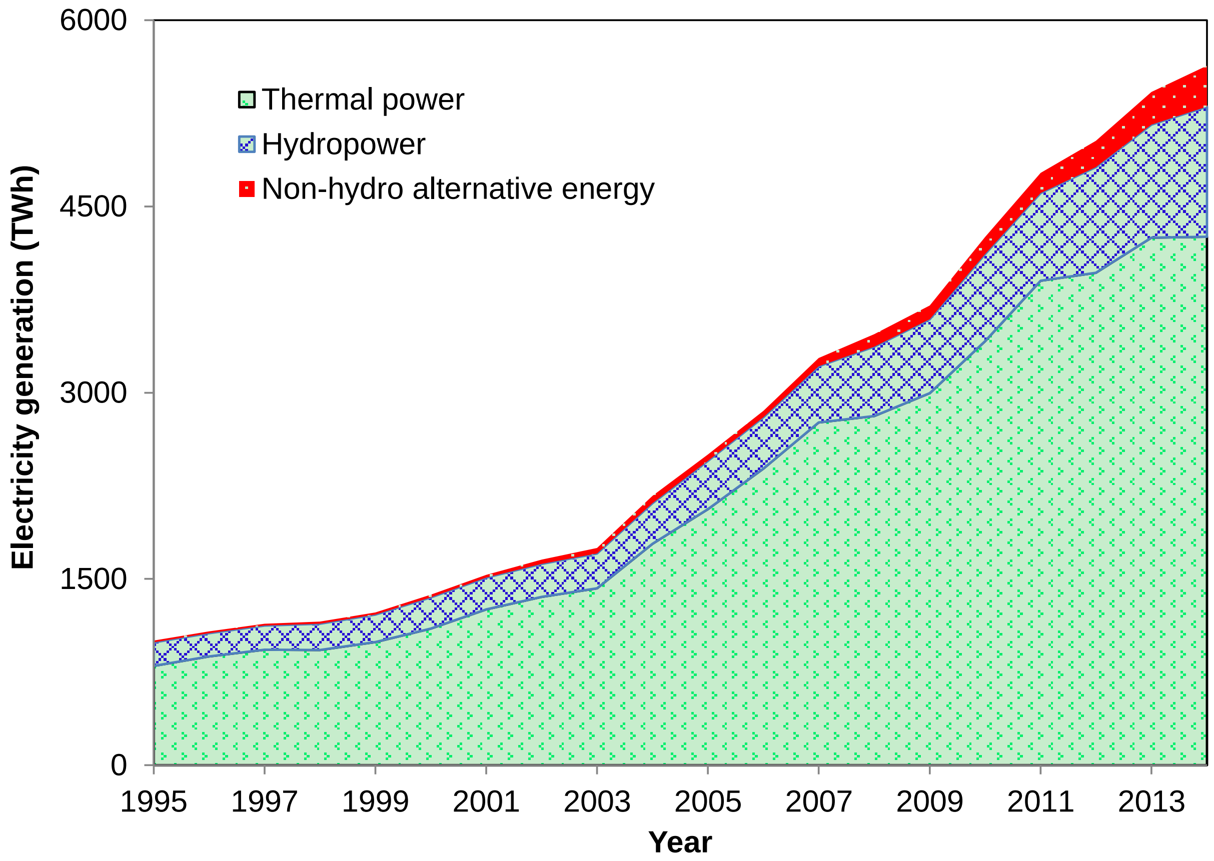

Supplementary Figure 2 | Growth of electricity generation in China over the period of 1995-2014 and the breakdown by generation sources. Data from the China Energy Statistical Yearbooks (http://tongji.cnki.net/kns55/Navi/NaviDefault.aspx).

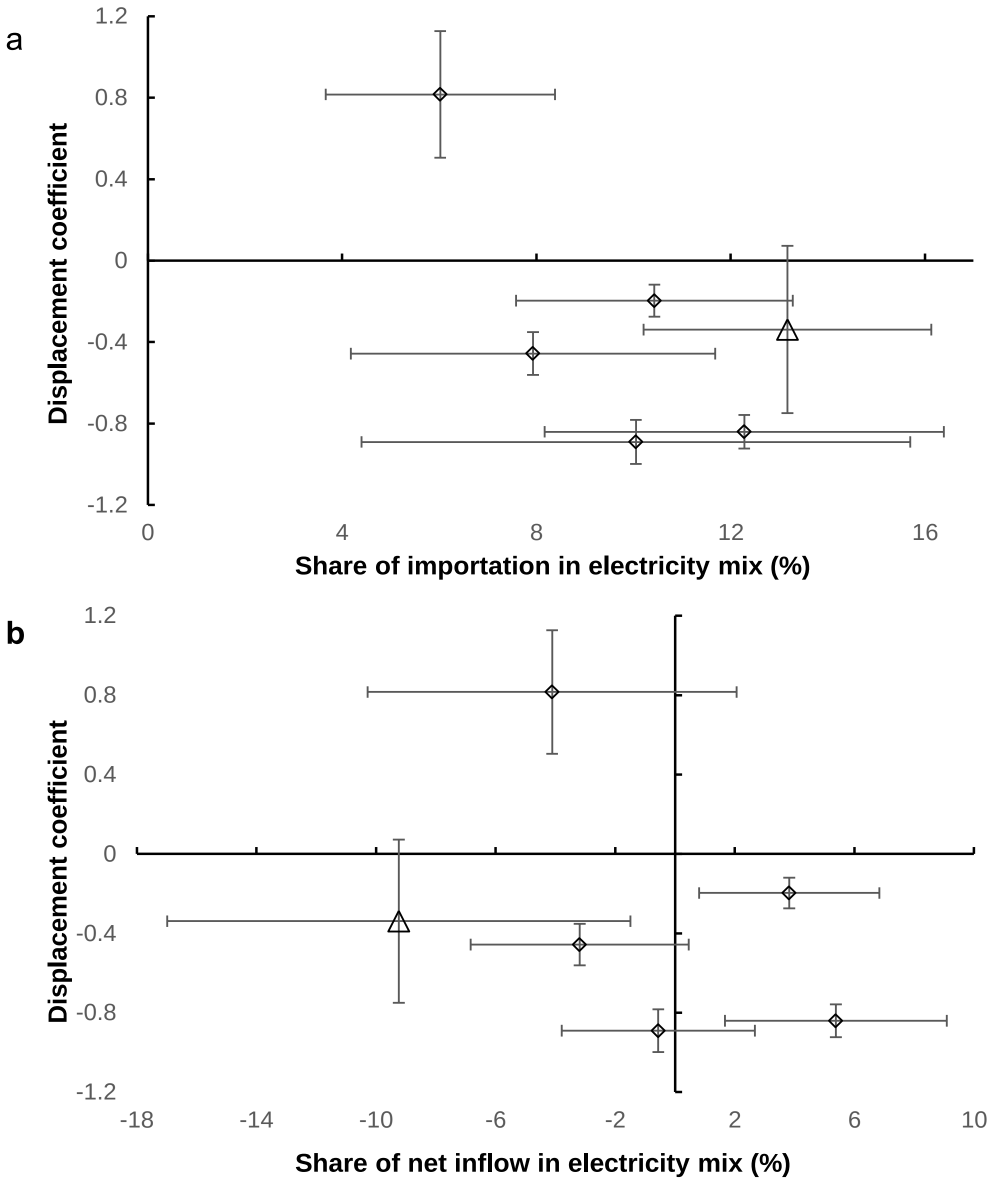

Supplementary Figure 3 | Displacement effect of trans-provincial imported electricity on local power generation. Shown are the displacement coefficients of trans-provincial important electricity for substituting fossil-fuel-generated electricity by local plants (within the provinces) as a function of (a) share of importation and (b) share of net inflow in the electricity supply mix of China's six inter-provincial regional power grids. Error bars represent standard errors of mean, and statistically significant and insignificant displacement coefficients (compared to 0, p-value < 0.05, two-tailed test) are plotted with the symbols of "◊" and "Δ", respectively.

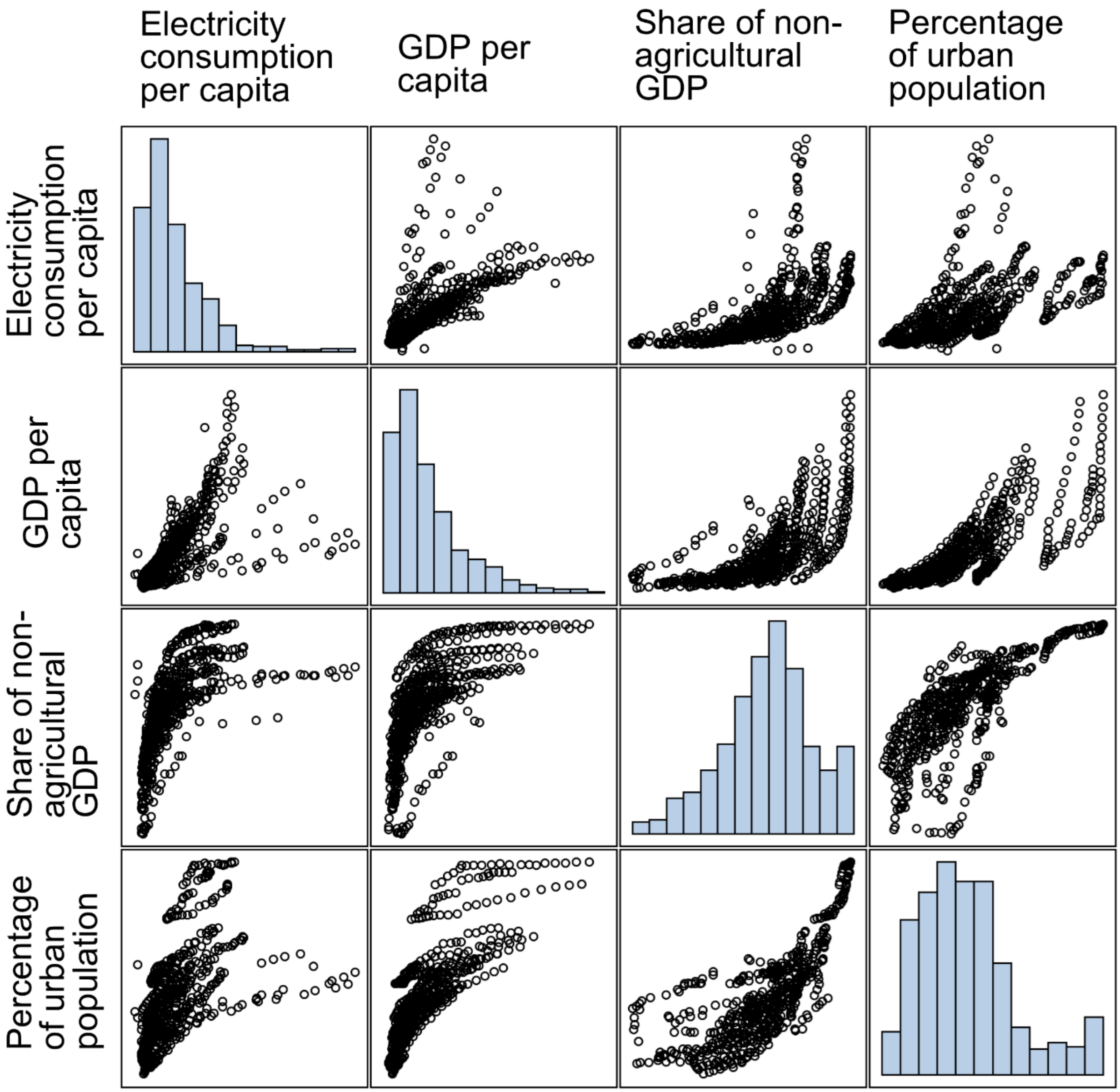

Supplementary Figure 4 | Correlations among electricity consumption and the model predictor variables. Shown is the scatter plot matrix depicting the correlations among the electricity consumption per capita, GDP per capita, share of non-agricultural GDP, and percentage of urban population across 30 provinces and municipalities in China over the period of 1995-2014.

Supplementary Table 1 | Advantages and limitations of major electricity generation sources. Shown is a comparison of the general performance, advantages, limitations, and future development in China for coal-fired power generation, hydropower, nuclear power, and solar energy and wind power.

Generation source	General performance	Advantages	Limitations	Development in the near future
Coal-fired generation	➢ Coal plants take days to hours to start up or adjust their output;<br>➢ Coal plants serve primarily as the baseload electricity generators and operate continuously at close to their rated capacities.	➢ With large reserves of coal and stable price, coal electricity is inexpensive and reliable compared to other forms of energy;<br>➢ The sizes of coal-fired plants are flexible, and they can be built anywhere with access to coal.	➢ Electricity production by coal-fired plants cannot be adjusted flexibly for the peak and off-peak consumption, resulting in surplus electricity that may not be put to productive uses or stored;<br>➢ To reduce coal transport, large number of coal-fired power plants have been constructed right near the major coal mines in northern and western China, which requires long-distance transmission networks to transmit the electricity to the load centers in the coastal provinces.	➢ China aims to reduce the reliance on coal-fired generation, even though growth in coal electricity is expected in the short term;<br>➢ The Energy Development Strategy Action Plan (2014-2020) unveiled recently sets the goal of reducing the share of coal in the primary energy mix to <62% and capping the coal consumption at 4.2 billion tonnes by 2020[1].
Hydropower	➢ Hydropower is a more reliable and affordable source of electricity than fossil-fuel-fired generation;<br>➢ Hydropower facilities are typically operated as baseload and dispatchable sources.	➢ Hydropower facilities can go from zero power to maximum output rapidly;<br>➢ Hydropower is particularly good at meeting the short-term variations in electricity demand occurring throughout the day.	➢ The dispatchability of hydropower is often limited by the water in the reservoirs: hydropower typically functions as baseload and dispatchable sources in the wet season, while it may be used mostly for matching the peak demand in the dry season;<br>➢ Large hydropower facilities are mostly located in the mountainous and remote areas, which requires long-distance transmission networks to transmit the electricity to the load centers.	➢ The remaining untapped hydropower resources in China are mainly located in the mountainous terrain, and are difficult to harness;<br>➢ The installed capacity of hydropower is expected to increase by 10-15 GW per year to raise the share of non-fossil fuels in China's energy mix to 15% by 2020[2].

Nuclear power	➢ Nuclear power is much more cost-effective and stable compared to the other non-hydro alternative energy options;<br>➢ Nuclear power is used primarily as a baseload source of electricity generation for both economic and technical/engineering reasons.	➢ With low variable cost, nuclear power readily replaces coal-fired generation at providing baseload power;<br>➢ Nuclear reactors can be built near the demand centers, as long as sufficient cooling water is available, avoiding long-distance transmission.	➢ Nuclear power plants have very high capital costs and the construction process may take several years or longer.	➢ China's latest Energy Development Strategy Action Plan (2014-2020) aims to increase the total installed capacity of nuclear power from the current level of slightly over 19 GW to 58 GW with an additional 30 GW generation capacity under construction by 2020[1].
Solar energy and wind power	➢ Solar energy and wind power are inherently weather-dependent and variable;<br>➢ The supply of solar energy and wind power usually does not follow the typical demand curve[3,4];<br>➢ Such intermittent sources cannot readily substitute coal-fired plants at supplying baseload.	➢ Although the initial cost of installation is relatively high, the cost of solar and wind electricity generation is practically zero;<br>➢ The maintenance requirement for solar and wind electricity generation is also relatively low.	➢ Solar energy and wind power are not continuously available and cannot be dispatched reliably to meet the electricity demand on the grid[3-6];<br>➢ Integrating solar energy and wind power into the power grid requires adequate, flexible sources of generation to smooth out the variations in their energy output[7,8];<br>➢ Large solar and wind energy sources are mostly located in western and northwestern China, necessitating long-distance transmission networks to transmit the electricity to the load centers in the coastal regions.	➢ The installed wind power capacity has reached 62.4 GW in 2011, and continued expansion of wind power is expected due to its cost competitiveness compared to other renewable energy sources[2];<br>➢ The installed solar power generation capacity (860 MW) represented <0.1% of China's electricity capacity by the end of 2010[2];<br>➢ With fast development in solar technology, a target of 20 GW installed capacity by 2020 has been set for solar energy[9].

Supplementary Table 2 | Full model parameters for the displacement effect of alternative energy and trans-provincial transported electricity in China estimated with models 1-3. Panel analyses were conducted using data from 30 provinces and municipalities during 1995-2014. Parameters for models on the displacement effect of alternative energy on fossil-fuel-generated electricity and that of the trans-provincial electricity transmission in China with combinations of GDP per capita, percentage of urban population, and share of non-agricultural GDP as the predictor variables were estimated. The cross-sectional and time-series effects were included in all panel models (results not shown). Although the coefficients of displacement, electricity importation and exportation estimated in all three models are close, the corresponding values for GDP per capita are significantly different due to the existence of strong correlations among the predictor variables of GDP per capita, percentage of urban population, and share of non-agricultural GDP (Supplementary Table 13).

Predictor variable	Model 1	Model 2	Model 3
Alternative energy per capita	-0.231*<br>(0.078)<br>[0.003]	-0.221*<br>(0.079)<br>[0.005]	-0.241*<br>(0.075)<br>[0.001]
Trans-provincial imported electricity per capita	-0.312*<br>(0.108)<br>[0.004]	-0.313*<br>(0.106)<br>[0.003]	-0.235*<br>(0.104)<br>[0.024]
Trans-provincial exported electricity per capita	1.497*<br>(0.151)<br>[<0.001]	1.512*<br>(0.152)<br>[<0.001]	1.459*<br>(0.149)<br>[<0.001]
GDP per capita	0.189*<br>(0.029)<br>[<0.001]	0.157*<br>(0.026)<br>[<0.001]	0.142*<br>(0.023)<br>[<0.001]
(GDP per capita)^2	-0.002*<br>(0.000)<br>[<0.001]	-0.001*<br>(0.000)<br>[<0.001]	-0.001*<br>(0.000)<br>[<0.001]
Percentage of urban population		5.223*<br>(1.386)<br>[<0.001]	4.880*<br>(1.333)<br>[<0.001]
Share of non-agricultural GDP			6.681*<br>(2.488)<br>[0.007]

* Statistically significant at the 0.05 alpha level (two-tailed test), standard errors are reported in parentheses, while *p*-values are presented in brackets.

Supplementary Table 3 | Model parameters for the displacement effect of alternative energy on the global scale. Panel analyses were conducted using data from 133 countries and regions, and the electricity demand was modeled as controlled by GDP per capita. Parameters for models on the displacement effect of alternative energy on fossil-fuel-generated electricity worldwide over the periods of 1960-2009 and 1995-2013 were estimated. The results obtained in the study of York[10], where the average displacement efficiency of alternative energy on fossil-fuel-generated electricity in a total of 132 countries and regions over the period of 1960-2009 was analyzed by modeling the electricity demand as controlled by GDP per capita, are also included for comparison. Both studies used similar models and the data from the same source. The global average displacement efficiency of alternative energy on fossil-fuel-generated electricity during 1995-2013 was analyzed to make comparison with that in China over the same period (global electricity use data in 2014 is not available from the database of World Bank yet at the present time).

Predictor variable	This study		York's study[10]
	1960-2009	1995-2013	1960-2009
Alternative energy per capita	-0.136* (0.020) [<0.001]	-0.114* (0.018) [<0.001]	-0.089* (0.009)
GDP per capita	0.139* (0.018) [<0.001]	0.154* (0.017) [<0.001]	296.453* (30.828)
(GDP per capita)^2	0.000* (0.000) [<0.001]	0.000* (0.000) [<0.001]	-8.044* (1.147)

The cross-sectional and time-series effects are included in all panel models but are not shown here.

* Statistically significant at the 0.05 alpha level (two-tailed test), standard errors are reported in parentheses, while *p*-values are presented in brackets.

Supplementary Table 4 | Full model parameters for the displacement effect of alternative energy and trans-provincial transported electricity in China's six regional grids. Panel analyses were conducted using data from 30 provinces and municipalities during 1995-2014, and the electricity demand was modeled as controlled by GDP per capita. Parameters for models on the displacement effect of alternative energy on fossil-fuel-generated electricity and that of trans-provincial electricity transmission in the six inter-provincial regional power grids of China were estimated.

Predictor variable	Inter-provincial regional power grid					
	East	Central	North	Northeast	Northwest	South
Alternative energy per capita	-0.709*<br>(0.142)<br>[<0.001]	-0.672*<br>(0.065)<br>[<0.001]	3.638*<br>(1.267)<br>[0.004]	3.960*<br>(0.608)<br>[<0.001]	-0.488*<br>(0.099)<br>[<0.001]	-0.888*<br>(0.072)<br>[<0.001]
Trans-provincial imported electricity per capita	-0.197*<br>(0.078)<br>[0.012]	-0.457*<br>(0.105)<br>[<0.001]	-0.841*<br>(0.083)<br>[<0.001]	-0.339<br>(0.411)<br>[0.409]	0.816*<br>(0.311)<br>[0.009]	-0.891*<br>(0.108)<br>[<0.001]
Trans-provincial exported electricity per capita	0.948*<br>(0.275)<br>[0.001]	0.608*<br>(0.081)<br>[<0.001]	1.883*<br>(0.166)<br>[<0.001]	0.306*<br>(0.124)<br>[0.013]	1.896*<br>(0.205)<br>[<0.001]	1.006*<br>(0.115)<br>[<0.001]
GDP per capita	0.246*<br>(0.022)<br>[<0.001]	0.036<br>(0.027)<br>[0.177]	0.142*<br>(0.023)<br>[<0.001]	0.898*<br>(0.116)<br>[<0.001]	-1.061*<br>(0.200)<br>[<0.001]	0.048<br>(0.032)<br>[0.138]
(GDP per capita)^2	-0.002*<br>(0.000)<br>[<0.001]	0.000<br>(0.000)<br>[0.452]	-0.001*<br>(0.000)<br>[<0.001]	-0.009*<br>(0.001)<br>[<0.001]	0.029*<br>(0.005)<br>[<0.001]	0.000<br>(0.000)<br>[0.956]

* Statistically significant at the 0.05 alpha level (two-tailed test), standard errors are reported in parentheses, while *p*-values are presented in brackets.

Supplementary Table 5 | Summary of the percentages of fossil-fuel-generated electricity and alternative energy produced in China's six regional grids. Shown is the descriptive statistics on the shares of fossil-fuel-generated electricity and alternative energy produced locally (within the provinces) in the electricity supply mix of China's six inter-provincial regional power grids between 1995 and 2014.

Grid	Share of fossil-fuel-generated electricity (%)				Share of alternative energy (%)			
	Mean	Std	Min	Max	Mean	Std	Min	Max
East	85.14	2.92	78.28	89.96	11.04	0.91	8.97	12.92
Central	63.78	3.67	56.96	71.11	39.41	3.40	34.57	47.43
North	93.38	4.38	84.91	99.31	1.24	1.03	0.18	3.70
Northeast	101.95	6.85	85.94	111.91	7.29	3.84	2.34	16.20
Northwest	79.75	6.23	68.29	91.71	24.36	2.53	18.83	28.57
South	64.94	3.87	55.35	71.12	35.62	6.24	25.92	46.23

Supplementary Table 6 | Summary of the percentage of electricity transmission in China's six regional grids. Shown is the descriptive statistics on the share of trans-provincial transported electricity (regardless of the source of generation) in the electricity supply mix of China's six inter-provincial regional power grids between 1995 and 2014.

Grid	Percentage of importation				Percentage of exportation				Percentage of net inflow			
	Mean	Std	Min	Max	Mean	Std	Min	Max	Mean	Std	Min	Max
East	10.43	2.85	5.96	16.13	6.60	0.85	5.25	8.45	3.82	3.01	0.26	10.46
Central	7.93	3.75	3.49	14.97	11.13	5.88	3.87	20.57	-3.19	3.65	-11.77	1.48
North	12.28	4.11	7.52	19.35	6.91	1.55	1.56	8.89	5.38	3.71	-0.01	11.57
Northeast	13.17	2.96	8.41	17.21	22.41	8.67	6.04	35.09	-9.24	7.75	-19.21	4.81
Northwest	6.02	2.36	1.13	10.39	10.14	6.49	1.69	22.55	-4.11	6.17	-15.61	3.55
South	10.05	5.65	1.90	19.41	10.62	5.10	5.28	20.99	-0.56	3.23	-5.30	5.84

Supplementary Table 7 | Full model parameters for the displacement effect of hydropower and non-hydro alternative energy in China. Panel analyses were conducted using data from 30 provinces and municipalities during 1995-2014, and the electricity demand was modeled as controlled by GDP per capita. Parameters for models on the displacement effect of hydropower and non-hydro alternative energy on fossil-fuel-generated electricity nationwide and in the six inter-provincial regional power grids, along with that of trans-provincial electricity transmission, were estimated.

Predictor variable	Nationwide	Inter-provincial regional power grid					
		East	Central	North	Northeast	Northwest	South
Hydropower per capita	-0.637*<br>(0.091)<br>[<0.001]	-1.168*<br>(0.117)<br>[<0.001]	-0.682*<br>(0.064)<br>[<0.001]	5.937*<br>(1.909)<br>[0.002]	1.160<br>(1.008)<br>[0.250]	-0.543*<br>(0.097)<br>[<0.001]	-0.896*<br>(0.073)<br>[<0.001]
Non-hydro alternative energy per capita	2.399*<br>(0.657)<br>[<0.001]	0.064<br>(0.206)<br>[0.756]	-1.251<br>(0.886)<br>[0.158]	3.167*<br>(1.358)<br>[0.020]	4.447*<br>(0.642)<br>[<0.001]	1.911*<br>(0.824)<br>[0.020]	-0.615<br>(0.575)<br>[0.285]
Trans-provincial imported electricity per capita	-0.271*<br>(0.106)<br>[0.011]	-0.180*<br>(0.075)<br>[0.016]	-0.464*<br>(0.105)<br>[<0.001]	-0.772*<br>(0.087)<br>[<0.001]	-0.170<br>(0.347)<br>[0.625]	0.532<br>(0.338)<br>[0.116]	-0.886*<br>(0.109)<br>[<0.001]
Trans-provincial exported electricity per capita	1.130*<br>(0.159)<br>[<0.001]	0.997*<br>(0.272)<br>[<0.001]	0.625*<br>(0.081)<br>[<0.001]	1.755*<br>(0.138)<br>[<0.001]	0.326*<br>(0.127)<br>[0.011]	1.756*<br>(0.199)<br>[<0.001]	1.012*<br>(0.114)<br>[<0.001]
GDP per capita	0.059<br>(0.031)<br>[0.056]	0.227*<br>(0.021)<br>[<0.001]	0.034<br>(0.027)<br>[0.204]	0.134*<br>(0.021)<br>[<0.001]	0.791*<br>(0.108)<br>[<0.001]	-0.829*<br>(0.207)<br>[<0.001]	0.055<br>(0.032)<br>[0.086]
(GDP per capita)^2	-0.001*<br>(0.000)<br>[0.005]	-0.002*<br>(0.000)<br>[<0.001]	0.000<br>(0.000)<br>[0.387]	-0.001*<br>(0.000)<br>[0.010]	-0.008*<br>(0.001)<br>[<0.001]	0.027*<br>(0.005)<br>[<0.001]	0.000<br>(0.000)<br>[0.811]

* Statistically significant at the 0.05 alpha level (two-tailed test), standard errors are reported in parentheses, while *p*-values are presented in brackets.

Supplementary Table 8 | Summary of the percentages of hydropower and non-hydro alternative energy production in China's six regional grids. Shown is the descriptive statistics on the shares of hydropower and non-hydro alternative energy produced locally (within the provinces) in the electricity supply mix of China's six inter-provincial regional power grids between 1995 and 2014.

Grid	Percentage of hydropower				Percentage of non-hydro alternative energy			
	Mean	Std	Min	Max	Mean	Std	Min	Max
East	7.82	2.51	4.23	12.04	3.22	2.02	0.30	6.67
Central	39.10	3.22	34.54	46.38	0.31	0.48	0	2.03
North	0.59	0.37	0.08	1.30	0.66	1.05	0	3.27
Northeast	4.18	1.79	1.80	9.10	3.12	4.39	0.04	13.01
Northwest	22.80	2.74	18.05	28.37	1.56	2.39	0.00	8.66
South	28.97	5.72	19.75	38.74	6.65	1.01	4.83	8.69

Supplementary Table 9 | Ranking of China's 30 provinces and municipalities (excluding Tibet) based on average hydropower production per capita over the period of 1995-2014.

Rank	Province/ municipality	Hydropower production per capita (kWh $year^{-1}$)		Fossil-fuel-fired electricity generation per capita (kWh $year^{-1}$)	
		Mean	Std	Mean	Std
1	Qinghai	3435.8	2420.4	1282.0	650.1
2	Hubei	1261.0	776.7	888.9	469.4
3	Yunnan	1227.6	1136.0	628.9	396.3
4	Sichuan	987.8	787.1	465.9	193.1
5	Fujian	779.9	264.8	1586.0	1101.1
6	Guizhou	752.5	475.2	1641.6	1088.2
7	Gansu	684.1	333.3	1469.1	838.7
8	Guangxi	614.4	333.5	650.2	496.9
9	Hunan	473.6	197.2	655.3	392.6
10	Chongqing	390.5	208.7	831.4	390.9
11	Xinjiang	315.0	216.9	2033.2	1930.3
12	Zhejiang	248.7	72.5	2415.0	1341.1
13	Guangdong	221.5	56.3	1837.2	691.9
14	Jilin	215.0	93.8	1412.7	512.6
15	Ningxia	204.5	88.3	6173.9	5235.9
16	Hainan	198.1	54.6	1009.8	708.0
17	Jiangxi	181.3	60.6	789.6	468.4
18	Shaanxi	141.2	75.1	1712.0	1138.4
19	Liaoning	79.5	37.4	2024.7	794.5
20	Henan	72.3	58.5	1511.1	831.6
21	Shanxi	59.3	33.8	3775.6	2176.9

22	Inner Mongolia	40.6	32.2	5634.7	4400.5
23	Heilongjiang	37.5	15.4	1507.6	395.9
24	Anhui	27.2	16.3	1418.5	993.3
25	Beijing	23.8	27.1	1312.2	144.6
26	Hebei	9.0	6.1	1938.6	784.6
27	Jiangsu	4.1	4.6	2720.6	1535.7
28	Shandong	0.9	1.2	2115.6	1035.5
29	Tianjin	0.5	0.7	3066.8	1023.1
30	Shanghai	0.0	0.0	3609.0	384.2

Supplementary Table 10 | Full model parameters for the displacement effect of hydropower and non-hydro alternative energy in the provinces with different hydropower production capacities. Panel analyses were conducted using data from 30 provinces and municipalities during 1995-2014, and the electricity demand was modeled as controlled by GDP per capita. Parameters for models on the displacement effect of hydropower and non-hydro alternative energy on fossil-fuel-generated electricity in the provinces ranked among the top and bottom halves in hydropower production per capita, along with that of trans-provincial electricity transmission, were estimated. Overall ranking of the 30 provinces and municipalities in hydropower production per capita during 1995-2014 is listed in Supplementary Table 9.

Predictor variable	Provinces with hydropower production per capita ranked in	
	Top half	Bottom half
Hydropower per capita	-0.573*<br>(0.092)<br>[<0.001]	1.133<br>(0.901)<br>[0.209]
Non-hydro alternative energy per capita	2.684*<br>(0.673)<br>[<0.001]	2.646*<br>(0.671)<br>[<0.001]
Trans-provincial imported electricity per capita	-0.049<br>(0.162)<br>[0.764]	-0.619*<br>(0.108)<br>[<0.001]
Trans-provincial exported electricity per capita	1.347*<br>(0.198)<br>[<0.001]	0.861*<br>(0.187)<br>[<0.001]
GDP per capita	-0.067<br>(0.036)<br>[0.065]	0.104*<br>(0.018)<br>[<0.001]
(GDP per capita)^2	0.001*<br>(0.000)<br>[0.042]	-0.001*<br>(0.000)<br>[<0.001]

* Statistically significant at the 0.05 alpha level (two-tailed test), standard errors are reported in parentheses, while *p*-values are presented in brackets.

Supplementary Table 11 | Advantages and environmental and human health impacts of major energy sources for electricity production. Shown is a comparison of the major advantages of fossil fuels, nuclear power, and renewable energy sources, along with their negative impacts on the environment and human health.

Energy source	Major advantages	Major negative impacts on the environment and human health
Fossil fuels (coal, oil, and natural gas)	➢ Fossil fuels are easy to find with abundant supply;<br>➢ Fossil fuels can be excavated at the reserves, processed at separate locations, and transported to energy users relatively easily;<br>➢ Power plants operating on fossil fuels can be constructed in almost any locations with access to large quantities of fuels (and cooling water);<br>➢ Fossil fuel-fired power generation is very cost-effective.	➢ Combustion of fossil fuels emits CO_2, which is a major contributor to global climate change and poses potentially catastrophic incremental climate change risk;<br>➢ Combustion of fossil fuels (particularly coal) may also release a range of air pollutants, such as particulate matter, polycyclic aromatic hydrocarbons (PAHs), SO_2, and NO_X, which can cause moderate to severe air pollution;<br>➢ Extraction of fossil fuels, particularly coal, affects wide areas of land, and can be detrimental, even disastrous, to the environment;<br>➢ Underground mining of coal is inherently dangerous and may endanger the lives of miners;<br>➢ Significant environmental hazards may result from oil spills during the extraction and transportation of crude oil;<br>➢ Serious water pollution often occurs at coal mines, while some oil fields can also have serious water pollution;<br>➢ Fossil-fuel-fired power generation has enormous environmental consequences, and it is a key contributor to air pollution, which poses risk to human health.
Nuclear power	➢ Nuclear power generation does not release CO_2, particular matter, or other gaseous pollutants, thus barely contributes to global warming or air pollution;<br>➢ Nuclear power plants can be built anywhere with access to large quantities of cooling water;<br>➢ Nuclear power generation requires very small mass of fuel, which significantly reduces the costs associated with the extraction, handling, and transportation of nuclear fuel (it should be noted that being radioactive, handling and transportation of the fuel is costly);<br>➢ Electricity generation from nuclear power plants is	➢ Although the volume of waste produced from nuclear power plants is small, management of nuclear waste is very difficult and expensive, and it takes very long time to eliminate its radioactivity and risk;<br>➢ With many components and parts being radioactive, decommissioning of nuclear power plants is expensive and takes many years;<br>➢ The mining, milling, and processing of nuclear fuel often produces serious water pollution, and potentially serious water pollution can also occur at the disposal sites of nuclear waste;<br>➢ Potential catastrophic accidents can occur at nuclear plants in the events of mismanagement or natural disasters (as exemplified by Chernobyl and Fukushima), while they are also potential targets of terrorist attacks;<br>➢ Nuclear accidents can have long-lasting effects over large regions,

	continuous and reliable (no dependence on natural aspects);<br>➢ Nuclear power plants typically have large generation capacities, and can fully operate for almost 90% of annual time.	resulting in releases of large amounts of radioactive particles into the environment and sickness and even deaths of people exposed to nuclear radiation.
Hydropower	➢ Hydropower generation does not directly emit greenhouse gases or air pollutants;<br>➢ Hydropower is much more predictable and reliable than wind and solar power (less reliable compared to coal-fired generation and nuclear power);<br>➢ With low operating and maintenance costs, hydroelectricity is inexpensive;<br>➢ Hydropower plants can have variable sizes, with the smaller ones having less ecological impact;<br>➢ Hydropower plants can be operated as pumped hydro storage for storing the power generated from intermittent renewable energy sources.	➢ Construction of large dams often causes relocation of populations;<br>➢ Significant changes in the landscape and ecosystems occur with reservoir flooding, which destroys the natural environment and habitat of animals;<br>➢ The normal river water flow is completely altered, which affects the water quality and fishes;<br>➢ Retention of sediments behind the dam accelerates the erosion of downstream river banks;<br>➢ Failures of large dammed-hydro facilities holding huge volumes of water due to natural disasters or terrorist attacks can cause catastrophic disasters to the downstream settlements and infrastructure.
Solar energy	➢ Solar energy is free and indefinitely renewable;<br>➢ Solar power generation does not emit greenhouse gases or cause air pollution;<br>➢ Solar panels can be installed in remote areas, where connecting to the regular power grid is too difficult or expensive.	➢ Solar power generation requires large areas of land, which may affect the wildlife;<br>➢ Manufacturing solar cells uses chemicals and energy, and releases hazardous waste materials that can contaminate water resources;<br>➢ Containing toxic metals, the end-of-life photovoltaic solar panels pose a future recycling and disposal problem.
Wind power	➢ Wind energy is free and can be captured efficiently by modern technology;<br>➢ No greenhouse gas or other air pollutant is produced during the generation of wind power;<br>➢ Lands below the wind turbines can still be used effectively for farming;<br>➢ Wind turbines require relatively low maintenance, and have low running costs;<br>➢ Wind turbines, which are available in varying sizes, can be used to supply electricity in remote areas without grid connection.	➢ Some pollution is produced during the manufacturing of wind turbines, while their installation is expensive;<br>➢ The turbine blades produce considerable noise, and wind turbines visually change local landscape;<br>➢ Large wind farms are needed to generate an adequate supply of wind energy;<br>➢ Spinning blades of wind turbines pose a threat to wildlife, and can cause injury or even deaths of birds and bats.
Biomass energy	➢ Biomass is a carbon neutral form of energy;<br>➢ Combustion of biomass produces lower levels of SO_2 compared to fossil fuels;<br>➢ Biomass products are abundant and their use in	➢ Although relatively clean compared to fossil fuels, electricity generation fueled by biomass still emits air pollutants, including particulate matter, CO, SO_2, and NO_X, although at much lower levels;<br>➢ Large areas of land and significant quantities of water are required for

	energy generation reduces the burden of landfills;<br>➢ Energy crops can be farmed and managed effectively, making biomass energy sustainable.	production of some energy crops, which can destroy species habitats and cause depletion of organic matter and nutrients from the soils.
Geothermal energy	➢ Geothermal energy is sustainable and free;<br>➢ Power generation based on geothermal energy has much lower environmental impact compared to fossil-fuel-fired generation;<br>➢ Geothermal energy allows constant, uninterrupted electricity generation.	➢ Production of geothermal power can release H_2S, CO_2, NH_3, and CH_4, which cause air and/or water pollution;<br>➢ Geothermal power plant operation also produces sludge containing silica and toxic heavy metals, which can be difficult to dispose of;<br>➢ Geothermal exploration may cause seismic instability, and the resulting minor earthquakes can cause building damages.

Supplementary Table 12 | Correlation between GDP and energy consumption in China's six regional grids. The Granger causality test was conducted for data of GDP and energy consumption in China's six trans-provincial regional power grids over the period of 1995-2014.

Grid	Null hypothesis	Chi-square	*p*-value
East	Increase in energy consumption caused GDP growth but GDP growth did not cause energy consumption increase	0	0.988
East	GDP growth caused energy consumption increase but increase in energy consumption did not bring GDP growth	23.21	<0.001
Central	Increase in energy consumption caused GDP growth but GDP growth did not cause energy consumption increase	1.71	0.190
Central	GDP growth caused energy consumption increase but increase in energy consumption did not bring GDP growth	7.02	0.008
North	Increase in energy consumption caused GDP growth but GDP growth did not cause energy consumption increase	6.58	0.010
North	GDP growth caused energy consumption increase but increase in energy consumption did not bring GDP growth	0.11	0.744
Northeast	Increase in energy consumption caused GDP growth but GDP growth did not cause energy consumption increase	13.24	<0.001
Northeast	GDP growth caused energy consumption increase but increase in energy consumption did not bring GDP growth	0.68	0.410
Northwest	Increase in energy consumption caused GDP growth but GDP growth did not cause energy consumption increase	9.02	0.003
Northwest	GDP growth caused energy consumption increase but increase in energy consumption did not bring GDP growth	0.25	0.615
South	Increase in energy consumption caused GDP growth but GDP growth did not cause energy consumption increase	2.40	0.122
South	GDP growth caused energy consumption increase but increase in energy consumption did not bring GDP growth	18.90	<0.001

Supplementary Table 13 | Correlations among energy consumption and the model predictor variables. Shown is the Pearson's correlation matrix for electricity consumption per capita, GDP per capita, share of non-agricultural GDP, and percentage of urban population across 30 provinces and municipalities in China based on the relevant data over the period of 1995-2014.

Variable	Electricity consumption per capita	GPD per capita	Share of non-agricultural GDP	Percentage of urban population
Electricity consumption per capita	1			
GPD per capita	0.630*	1		
Share of non-agricultural GDP	0.609*	0.689*	1	
Percentage of urban population	0.524*	0.805*	0.770*	1

* Statistically significant at the 0.05 alpha-level (two-tailed test).

Supplementary References

1 State Council. *The Energy Development Strategy Action Plan (2014-2020)*. www.gov.cn/zhengce/content/2014-11/19/content_9222.htm, 2014.

2 Hu, Y. & Cheng, H. Development and bottlenecks of renewable electricity generation in China: A critical review. *Environ. Sci. Technol.* **47**, 3044-3056 (2013).

3 Yang, M., Patino-Echeverri, D. & Yang, F. Wind power generation in China: Understanding the mismatch between capacity and generation. *Renew. Energ.* **41**, 145-151 (2012).

4 Zhang, S. & He, Y. Analysis on the development and policy of solar PV power in China. *Renew. Sust. Energ. Rev* **21**, 393-401 (2013).

5 Wang, Q. Effective policies for renewable energy-the example of China's wind power-lessons for China's photovoltaic power. *Renew. Sust. Energ. Rev* **14**, 702-712 (2010).

6 Wang, Z., Qin, H. & Lewis, J. I. China's wind power industry: Policy support, technological achievements, and emerging challenges. *Energ. Policy* **51**, 80-88 (2012).

7 Barton, J. P. & Infield, D. G. Energy storage and its use with intermittent renewable energy. *IEEE T. Energ. Conver.* **19**, 441-448 (2004).

8 Liao, H., Liu, D., Huang, Y., Chen, Y. & Liu, J. A study on compatibility of smart grid based on large-scale energy storage system. *Dianli Xitong Zidonghua* **34**, 15-19 (2010) (in Chinese).

9 Martinot, E. & Li, J. China's latest leap: an update on renewables policy. *Renewable Energy World* **13**, 51-57 (2010).

10 York, R. Do alternative energy sources displace fossil fuels? *Nat. Clim. Change* **2**, 441-443 (2012).
